# MS-YOLO: A Lightweight and High-Precision YOLO Model for Drowning Detection

**DOI:** 10.3390/s24216955

**Published:** 2024-10-30

**Authors:** Qi Song, Bodan Yao, Yunlong Xue, Shude Ji

**Affiliations:** 1 School of Automation, Shenyang Aerospace University, Shenyang 110136, China; yaobodan@stu.sau.edu.cn (B.Y.); xueyunlong@stu.sau.edu.cn (Y.X.); superjsd@163.com (S.J.); 2 State Key Laboratory of Robotics, Shenyang Institute of Automation, Chinese Academy of Sciences, Beijing 100045, China

**Keywords:** drowning detection, MS-YOLO, YOLOv8, lightweight high-accuracy model

## Abstract

A novel detection model, MS-YOLO, is developed in this paper to improve the efficiency of drowning rescue operations. The model is lightweight, high in precision, and applicable for intelligent hardware platforms. Firstly, the MD-C2F structure is built to capture the subtle movements and posture changes in various aquatic environments, with a light weight achieved by introducing dynamic convolution (DcConv). To make the model perform better in small object detection, the EMA mechanism is incorporated into the MD-C2F. Secondly, the MSI-SPPF module is constructed to improve the performance in identifying the features of different scales and the understanding of complex backgrounds. Finally, the ConCat single-channel fusion is replaced by BiFPN weighted channel fusion to retain more feature information and remove the irrelevant information in drowning features. Relative to the Faster R-CNN, SSD, YOLOv6, YOLOv9, and YOLOv10, the MS-YOLO achieves an average accuracy of 86.4% in detection on a self-built dataset at an ultra-low computational cost of 7.3 GFLOPs.

## 1. Introduction

As a major global public health issue, drowning leads to approximately 236,000 deaths annually, ranking as the third leading cause of unintentional injury-related deaths worldwide [1]. In order to reduce the number of deaths, it is essential to detect drowning incidents in a rapid and accurate way. In the past, the practice of drowning detection was completely reliant on manual observation due to technical reasons. However, there are limitations faced by manual observation, such as the restricted field of view, fatigue, and reduced attention over time. Consequently, there is a heightened possibility of missed or false alarms, by which the safety of swimmers and potential drowning victims is compromised [2,3,4].

As technology advances, video surveillance and wearable multi-sensor technology have been playing a critical role in the practice of drowning detection and rescue. Salehi et al. devised a method in which drowning is detected through HSV color space analysis [5]. However, the method shows sensitivity to lighting changes, which results in false or missed detection. Jalalifar et al. proposed a wearable self-rescue alarm system for children, which can be used to detect drowning early by monitoring heart rate, water depth, and immersion time [6]. Despite its advantages, there remain challenges in the use of acoustic signals in wearable devices, such as underwater multipath propagation, the reduced effectiveness of transmission, and the delay to detection [7]. Therefore, it is imperative to develop a more efficient and reliable system for autonomous drowning detection.

Nowadays, the rapid development of deep learning technology has contributed a novel and effective solution for automatic drowning detection which addresses the downsides of manual observation, wearable multi-sensor systems, and video surveillance technologies. Deep learning and video analysis technologies can be applied to develop an efficient drowning rescue solution. The model extracts features from the real-time video data captured by cameras to identify any potential abnormal behavior through time-series analysis. Once an anomaly is detected, the video analysis features are compared with the predefined drowning behavior characteristics, to issue alerts or trigger rescue operations in real time. This enables automatic monitoring and early warning, for more comprehensive and time-efficient drowning detection. Developed by Liu et al., the embedded AI device incorporates a camera and a waterproof casing to capture and analyze underwater video streams for detection of drowning incidents. Despite the effectiveness in reducing the impact of complex surface environments, it remains susceptible to interference from water quality, light obstructions, and open-water environments [8]. Kharrat et al. proposed neural networks with the ability to distinguish between drowning and standard swimming patterns [9]. However, the neural networks fall short of the expectations for performance in various complex environments, such as crowded pools, which necessitates improvement. Claesson et al. adopted online machine-learning algorithms to identify drowning victims [10]. Despite its effectiveness in open water, this approach compromises the speed of detection, which is deemed crucial for real-time monitoring. Alotaibi et al. proposed to detect and classify drowning by integrating the Internet of Things with transfer learning techniques [11]. Nevertheless, in practice, there may be considerable diversity in drowning situations and environments, and the possibility that transfer learning models are insufficient in generalization ability.

For high-accuracy and real-time performance in real-world rescue operations, it is necessary to ensure the light weight, accuracy, and generalization ability of drowning detection algorithms. Despite the high accuracy achieved by such two-stage algorithms as the R-CNN [12,13,14], SPP-Net [15], Fast R-CNN [16,17], and Faster R-CNN [18,19,20], real-time performance is requisite. To solve this issue, the one-stage algorithms represented by the SSD [21,22,23] and YOLO [24,25,26,27] can be applied. However, the SSD fails in balancing accuracy with speed. The YOLO series can achieve balance between efficient detection and high accuracy as it relies on iteration to enhance performance. Jensen et al. adopted YOLOv2 to detect drowning in pools [28]. In subsequent studies, improved systems were developed on the basis of YOLOv4 and YOLOv5 [29,30]. However, these models lag behind the latest technology and there is a decline in their research value.

In the present study, a novel algorithm named MS-YOLO is presented on the basis of the latest YOLOv8. By testing this algorithm on a custom dataset, results are obtained that indicate a significant improvement achieved by the MS-YOLO in detection accuracy and weight. The accuracy of the model reaches 86.4% at the model size of 7.3 GFLOPs. The main contributions of this paper are outlined as follows:

1. To capture the subtle movements and posture changes in various aquatic environments while enhancing the identification of drowning behavior, a self-designed dual-residual MD-C2F module is put forward. Through the combination with dynamic convolution (DcConv), model complexity is lowered. By integrating multi-scale attention (EMA), the MDE-C2F module improves the outcome of small object detection in complex environments.
2. A novel spatial pyramid structure (MSI-SPPF) based on SPPF is developed to enable the model to better understand the relationship between various aquatic backgrounds and drowning targets while reducing the false and missed detection caused by environmental factors.
3. To remove the irrelevant drowning features while emphasizing the critical characteristics, the feature fusion algorithm is improved using a bi-directional feature pyramid network (BiFPN).

## 2. Datasets

Allowing for the lack of publicly available datasets focusing on drowning incidents, a custom dataset was developed in this study by simulating drowning scenarios to evaluate how the MS-YOLO algorithm performs. In 2020, Carballo-Fazanes et al. published a study on drowning behaviors, with three primary manifestations of drowning identified: Instinctive Drowning Response (IDR), Climbing Ladder Motion, and Backward Stroke. Referred to as the involuntary actions of individuals, the Instinctive Drowning Response is characterized by the individual extending their arms laterally and pressing down on the water’s surface to breathe. The Climbing Ladder Motion is used to describe the efforts made by a drowning person to keep their head above water by performing ladder-climbing-like motions with their arms. The Backward Stroke involves vigorous splashing and arm rotations [31]. This dataset was constructed around these three manifestations to ensure accuracy and validity.

To simulate the scenarios of drowning while ensuring the diversity of the dataset, four volunteers with different figures, including slim, average, and strong, were recruited to reveal the variation in underwater movements between different figures. Prior to data collection, the volunteers were trained to ensure data quality. By referring to the aforementioned drowning behavior studies, three typical drowning behaviors were simulated to ensure the reliability of the drowning data in the dataset: Instinctive Drowning Response, ladder climbing action, and back-paddling.

**Figure 1:**
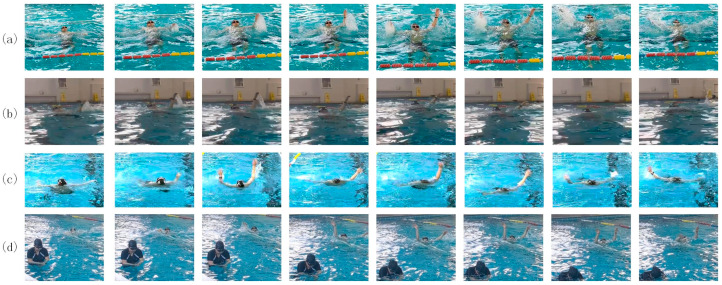
The scenarios of drowning from multiple perspectives: (**a**) aerial view, (**b**) water-level view, and (**c**,**d**) shoreline view. These datasets address the identification requirements of amphibious search and rescue systems, with various swimming postures and bystander safety behaviors incorporated to improve the model in terms of recognition accuracy.

In addition, an indoor swimming pool was selected for data collection. Under normal illumination and water conditions, the body movements and facial expressions of the drowning individuals are more easily identifiable. Due to the diverse backgrounds in the pool, with other swimmers engaging in backstroke, breaststroke, freestyle, water treading, and other playful movements, the diversity of dataset was enriched to better simulate the complex scenarios in the real world. Because of such diverse data, not only were drowning behaviors included in the custom-built dataset as positive samples, but other safe behaviors were also included as negative samples, which improved the regularization performance during model training. Furthermore, data were collected from three different perspectives, aerial, shore, and water-level views, thus enriching the data collected to comprise the dataset with varying intensities of lighting as much as possible. This was conducive to the simulation of drowning in different scenarios such as in daylight, at nighttime, under insufficient lighting, and with reflection on the water surface. See Figure 1.

**Figure 2:**
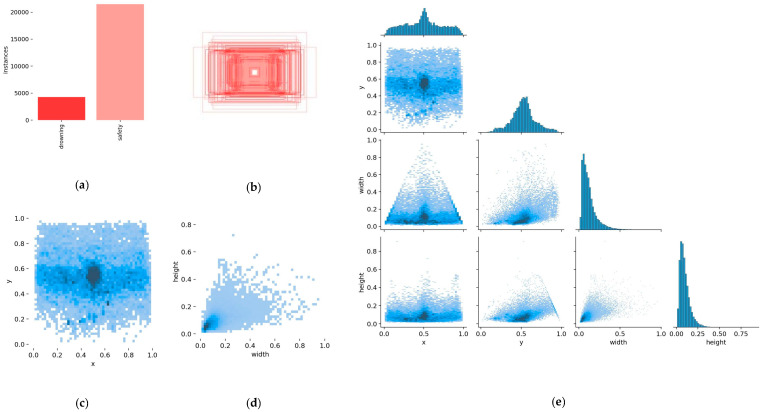
Dataset annotation file statistics and visualization. (**a**) Category number. (**b**) Distribution of central points. (**c**) Distribution of central points. (**d**) Width and height distribution. (**e**) Label distribution details.

For this paper, the dataset was captured using cameras and annotated with a labeling tool. Six thousand five hundred and ninety-four images were collected and divided on a random basis into training (5149 images) and validation sets (1445 images) at an 8:2 ratio. The images were classed into two categories: drowning and non-drowning. These annotated images were used for experimental analysis. Figure 2 shows the annotations and visualizations of the experiment results. In this figure, each matrix cell represents the labels used during model training, with the correlation between labels indicated by the intensity of the color. Darker cells indicate more robust learning of label correlations by the model, while lighter cells represent weaker correlations. Figure 2a displays a histogram of the number of samples per class. Figure 2b illustrates the lengths and widths of bounding boxes when the x and y values of all labels are set to the same position. Figure 2c depicts the distribution of x and y coordinates for the labels in the images. Figure 2d shows the aspect ratio of bounding box widths to heights. Figure 2e provides a detailed view of the label distribution within the original dataset. According to the analytical results, there was an uneven distribution of drowning incidents in the custom dataset. Meanwhile, the applicability of this method to drowning detection scenarios was confirmed by the precise positioning of the bounding boxes.

## 3. Methods

### 3.1. YOLOv8 Improved Model

**Figure 3:**
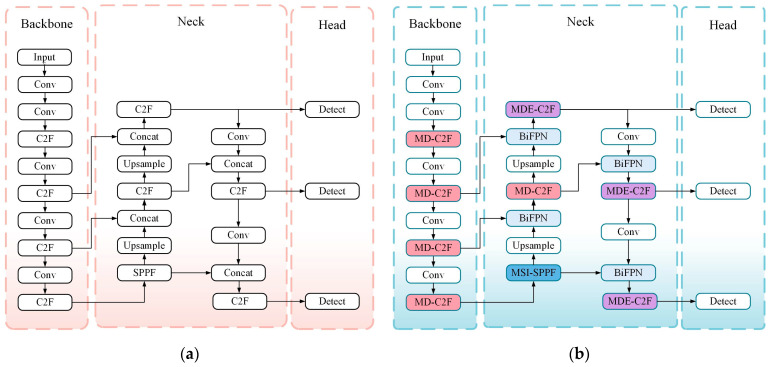
(**a**) The diagram shows the structure of YOLOv8. (**b**) The diagram shows the structure of MS-YOLO.

Herein, a novel algorithm is introduced. Named MS-YOLO, it is based on the YOLOv8 model constructed by Ultralytics. According to Figure 3a, the YOLOv8 model comprises four main components: the input module, backbone structure, Neck structure, and Head structure. The input module deals with adaptive image scaling, dimensional adjustments, and Mosaic data augmentation. Involving C2F and SPPF structures, the backbone module enhances gradient flow while enabling multi-scale feature integration. By incorporating a FPN and PAN, the Neck structure improves the outcome of feature fusion. The Head enhances performance through a decoupled head structure.

Despite the compact design and fast inference capabilities of YOLOv8, its performance remains unsatisfactory under complex scenarios. It performs poorly in the presence of inconsistent or overlapping object scales, producing unsatisfactory results. As shown in Figure 3b, the MS-YOLO model introduces new components to address this problem, including MD-C2F and MDE-C2F modules. Therefore, the outcome of feature extraction is enhanced, particularly for the detection of drowning behaviors and small objects. Moreover, the newly designed multi-scale spatial pyramid fusion module (MSI-SPPF) is involved to differentiate drowning behaviors from the background. With the BiFPN module in place, performance is improved by removing the irrelevant information via weighted feature fusion. Because of these enhancements, there is a significant increase in the overall accuracy of the model.

### 3.2. A Novel C2F Architecture

#### 3.2.1. MD-C2F

Actually, there are some tough challenges presented by drowning detection in practice. The changes in image angles often lead to water surface reflections, as a result of which the visibility of a drowning individual is obscured. Additionally, there are variations in drowning behaviors and characteristics between different aquatic environments.

Therefore, the detection algorithm must be applicable to capturing subtle movements and postures in different underwater environments and identifying drowning incidents accurately against complex backgrounds. To address the occurrence of occlusion and the complexity in backgrounds, Kerdvibulvech proposed a solution that combines an extended particle filter with a deterministic clustering algorithm. It can be relied on to identify hand movements in real time and accurately track fingertips even against complex backgrounds and at varying intensities of lighting [32]. Inspired by it, dynamic convolution (DcConv) is incorporated into the design of the MD-C2F module, which allows the real-time updating of features within the bottleneck structure to cope with environmental uncertainties.

To overcome these challenges, a novel MD-C2F module is developed in this paper. Distinct from conventional C2F structures, the MD-C2F combines dynamic convolution (DcConv) within its bottleneck architecture, which enables the model to perform better in feature extraction while reducing computational complexity significantly. In addition, the bottleneck residual structure and the top-down processing approach of the C2F module are refined. Thus, the model is more capable of extracting and representing multi-level features, which leads to a higher accuracy in the detection of drowning behaviors in different environments.

**Figure 4:**
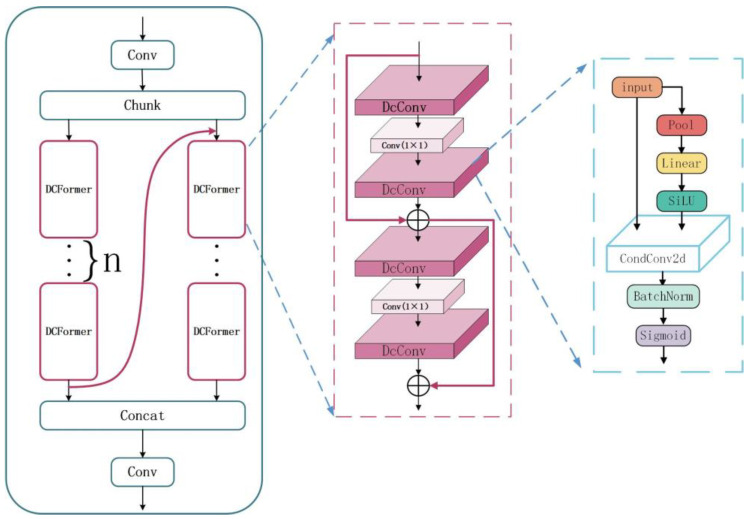
The structural diagram of MD-C2F.

The MD-C2F structure is illustrated in Figure 4. Dissimilar from the continuous residual structure of C2F structures, the MD-C2F groups input data, concatenating the output of the first DCFormer processing chain with the input of the second DCFormer processing chain. Then, the outputs of all DCFormer processing chains are concatenated. Under this architecture, the features are efficiently integrated and transmitted through multi-level convolution operations, which significantly improves the depth and breadth of feature extraction. Equations (1) and (2) illustrate the case where n = 2.

The calculation process of the MD-C2F structure is expressed as follows:

(1) $$\left\{\begin{array}{c} Y_{1},Y_{2}=Chunk(Conv(X),2)\\ Y_{1}^{\prime }=DCFormer(Y_{1})\\ Y_{2}^{\prime }=DCFormer(Cat(Y_{1}^{\prime },Y_{2})) \end{array}\right.$$

(2) $$Y=Conv(Cat(Y_{1}^{\prime },Y_{2}^{\prime }))$$

where two groups of data are obtained by splitting the input. “*Chunk*” refers to the operation of splitting the tensor, “*Conv*” denotes the convolution operation, “*Cat*” represents the feature concatenation operation, and *Y* stands for the final output of the module.

As an efficient component of convolutional neural networks, the DCFormer module consists of four DcConv layers and two standard convolutional layers. These layers are connected by a dual-layer residual structure. For each DcConv layer, the optimal convolutional kernel is selected in a flexible way, which enhances the efficiency and accuracy in feature extraction. The channels are adjusted by the standard convolutional layers. In comparison to the single residual structure in the bottleneck, the dual-layer connection is deepened and the process of feature extraction is refined.

As an innovative neural network unit, DcConv combines dynamic convolution, batch normalization, and activation functions. Additionally, it relies on adaptive average pooling to reduce dimensionality. DcConv selects kernels constantly based on weights. In this way, the convolutional operations are undertaken to make automatic adjustment, which improves the precision and flexibility in feature extraction.

The computational process of DCFormer is described as follows:

(3) $$\left\{\begin{array}{c} F_{1}=DcConv(Conv(DcConv(X)))\\ F_{2}=X+F_{1}\\ Y=F_{2}+DcConv(Conv(DcConv(F_{2}))) \end{array}\right.$$

(4) $$\left\{\begin{array}{c} P=Flatten(AdaptiveAvgPool2d(X,1))\\ R=\sigma (P\cdot W_{r}+b_{r})\\ V=\sum_{e=1}^{E}R_{e}\cdot (X*W_{e})\\ O=\sigma (BatchNorm2d(V)) \end{array}\right.$$

where *X* represents the input feature information; “*Flatten*” denotes the dimensionality reduction operation; “*AdaptiveAvgPool*” refers to the adaptive average pooling operation; *R* represents the expert weights for each sample; *W_r_* and *b_r_* denote the weights and biases of the fully connected layer, respectively; *R_e_* indicates the weight of the e expert; *W_e_* represents the convolutional kernel of the e expert; * denotes the convolution operation; *V* indicates the output obtained through dynamic convolution, which represents the Sigmoid activation function; “*BatchNorm2d*” refers to the batch normalization operation; and *O* represents the output of the DcConv.

#### 3.2.2. MDE-C2F

A significant breakthrough in computational methods is to introduce attention mechanisms. Because of their high efficiency, they are regarded as an effective solution for small object detection [33]. In the context of drowning detection, the limitations on viewing angle cause the difficulty in recognizing small objects. Ouyang et al. put forward the efficient multi-scale attention (EMA) module [34,35]. On this basis, the MD-C2F model is further improved. With the EMA module incorporated into the DCFormer, the MDE-C2F model is developed. Due to this enhancement, the lightweight nature of the model is maintained. Meanwhile, it performs significantly better in detecting small objects.

**Figure 5:**
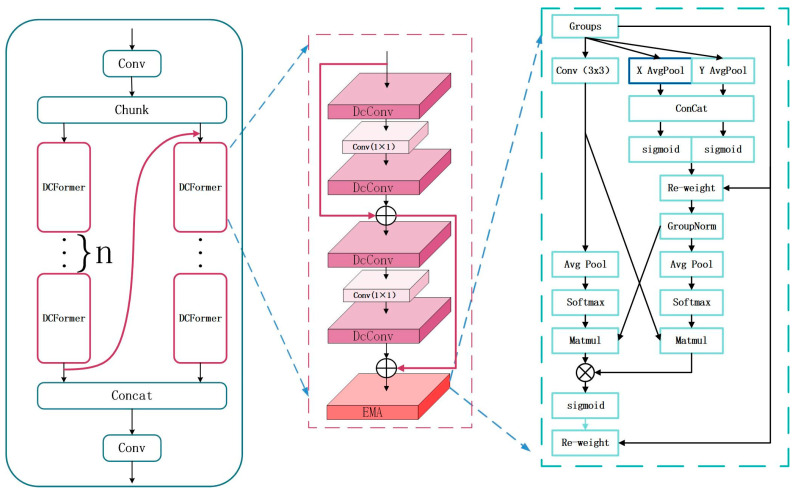
The MDE-C2F structure with EMA incorporated.

As an attention mechanism, the EMA module relies on channel grouping. During forward propagation, the input feature map is divided into two groups. The first group is subjected to adaptive average pooling, followed by fusion based on 1 × 1 convolution. It is then split and multiplied element-wise with the original feature map. Subsequently, group normalization is performed. The second group is also subjected to adaptive average pooling, with weights calculated using the Softmax function. Finally, the outputs of both groups are first weighted and then combined for the final output. Through channel grouping, the mechanism improves the performance in feature extraction. Moreover, feature map interaction is enhanced. This leads to an efficient attention mechanism. The pooling operation is presented as follows:

(5) $$\left\{\begin{array}{c} Z_{C}^{H}=\frac{1}{W}\sum_{0\leq i\leq W}x_{C}(H,i)\\ Z_{C}^{W}=\frac{1}{H}\sum_{0\leq j\leq H}x_{C}(j,W)\\ f=\sigma (\frac{1}{H\times W}\sum_{j}^{H}\sum_{i}^{M}x_{C}(i,j)) \end{array}\right.$$

(6) $$h_{c}(i,j)=x_{c}(i,j)\times f(w_{i})\times f(h_{j})$$

where $x_{C}$ represents the feature map with the input dimensions of *H × W × C*. Under the EMA mechanism, a pooling kernel of size *H × W* is used to encode the coordinates of each channel in the horizontal and vertical directions. $Z_{C}^{H}$ refers to the encoding result in the horizontal direction after a fixed height (H). Similarly, $Z_{C}^{W}$ indicates the encoding result in the vertical direction after a fixed width (W). f is the intermediate feature map of spatial information in the horizontal and vertical directions. × represents the weighting operation. $h_{C}$ refers to the weighted output of the intermediate feature map f and the input feature map $x_{C}$. See Figure 5.

#### 3.2.3. MSI-SPPF

In the context of drowning detection, what is crucial to reducing false positives and negatives lies in the relationship between the background and the target. False positives can be easily caused by reflections, ripples, or floating objects on the water surface. Additionally, it is possible that occlusion or murky conditions cause the targets in the water to be missed. Therefore, a Multi-Scale Spatial Pyramid Pooling (MSI-SPPF) structure is developed to address these issues. Apart from enhancing the understanding of complex backgrounds by the model, this structure also improves the accuracy of detection.

**Figure 6:**
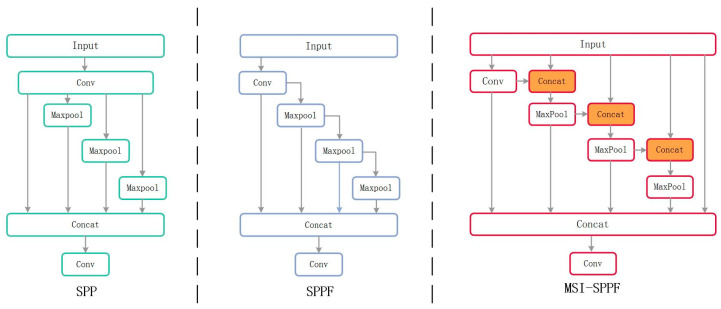
The structures of SPP, SPPF, and MSI-SPPF are presented.

In the research on object recognition, the Spatial Pyramid Pooling (SPP) module and its improved versions, such as the Fast Spatial Pyramid Pooling (SPPF) [36], Atrous Spatial Pyramid Pooling (ASPP) [37], and the pooling modules combined with Cross-Stage Partial Networks SPPCSPC [38,39] and SPPFCSPC [40], have been widely used in object detection algorithms. As shown in Figure 6, the pooling layers of different sizes are used by the SPP structure to extract features. However, independent processing may result in incompleteness of the information.

Despite the connections created by the SPPF structure between pooling layers, the original feature map is not integrated. This may result in information loss, leading to false or missed detection of drowning. To solve this problem, the MSI-SPPF structure is developed. This module starts by reducing the number of channels and computational complexity through a convolutional layer. Then, the output is concatenated with the original feature map. The feature map passes through max-pooling layers, and each pooled map is concatenated with the initial feature map. Finally, all pooled feature maps are combined into a multi-scale fused feature map. Thus, feature extraction and computational efficiency are significantly enhanced. The calculation of MSI-SPPF is expressed as Equations (7) and (8).

(7) $$\left\{\begin{array}{c} y_{1}=Conv(x)\\ y_{2}=Max(Cat(y_{1},x))\\ y_{3}=Max(Cat(y_{2},x))\\ y_{4}=Max(Cat(y_{3},x)) \end{array}\right.$$

(8) $$y_{out}=Conv(Cat(y_{1},y_{2},y_{3},y_{4},x))$$

where *x* represents the input features; “*Conv*” denotes the convolution operation; “*Max*” refers to the max-pooling operation; “*Cat*” indicates the feature concatenation operation; $y_{1},y_{2},y_{3},y_{4}$ refer to the output of each branch; and $y_{out}$ denotes the final output obtained.

#### 3.2.4. BiFPN

A major challenge in drowning detection is removing the irrelevant features while highlighting critical drowning-related information. However, the model may capture unnecessary features from complex backgrounds, such as water ripples and shadows. In addition to increasing computational complexity, this also causes interference with effective feature extraction. It is proposed to resolve this issue via BiFPN weighted feature fusion with the single-channel ConCat fusion replaced.

**Figure 7:**
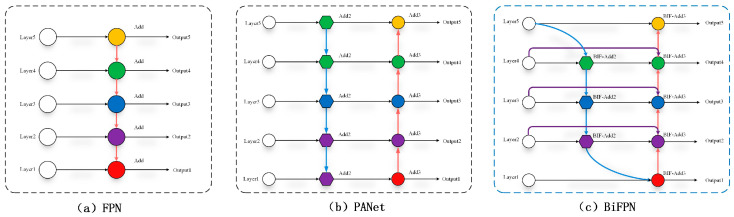
Structures of FPN, PANet, and BiFPN.

As shown in Figure 7a–c, the BiFPN is an evolution of the FPN and PANet. The FPN extracts multi-scale features, while the PANet incorporates bottom-up path aggregation [41]. However, there is a risk of losing feature information for both structures. In response, Chiley et al. presented the BiFPN structure. Subsequent to weighting, the BiFPN module concatenates the input features, which allows the more effective integration of information from different levels. Also, feature representation is enriched. The detailed information is retained under the weighted feature fusion strategy, which improves the model in terms of detection accuracy and robustness. This process is expressed as Equation (9).

(9) $$Out=\sum_{i}\frac{w_{i}I_{i}}{\varepsilon +\sum_{j}w_{j}}$$

where $I_{i}$ denotes the input features; *Out* indicates the result after weighted fusion; $w_{i}$ and $w_{j}$ refer to the learnable weights; and ε=0.0001 represents a small constant value needed for stable output.

## 4. Experiments and Discussion

### 4.1. Experimental Environment and Configuration

The experiments were conducted on a Linux platform with an NVIDIA GeForce RTX 3090 GPU and an AMD EPYC 7542 CPU as the hardware. The MS-YOLO model was implemented under the Pytorch 2.2.1 deep learning framework. The software environment included CUDA 12.1 and Python 3.9. The number of epochs for model training was set to 300, and the learning rate was set to 0.01. Below is a table showing the parameters of the experimental configuration.

For the improved reliability of the experiments, a series of tests was conducted using the original YOLOv8 model. Through repeated parameter adjustments, multiple experimental trials were performed. Finally, some key hyperparameters were standardized, with the same settings shown in Table 1 adopted for all experiments. To accelerate training, the batch size was set to 64, with an image size of 640 × 640 and a total of 300 epochs. The learning rate was set to 0.01, and SGD was taken as the optimizer. Additionally, the cache was enabled, with 16 workers configured to improve the efficiency of training, as detailed in Table 2.

### 4.2. Evaluation Metrics

For a thorough evaluation of how the proposed MS-YOLO model performs, the following performance metrics were adopted:

Precision: This metric reflects the performance of the model in identifying the relevant objects correctly. It is defined as the ratio of true positives to (*T_P_*) the sum of true positives and false positives (*F_P_*). The calculation formula is expressed as follows:

(10) $$P=\frac{T_{p}}{T_{P}+F_{P}}\times 100\%$$

Recall: Recall is reflective of the relationship present between the number of correctly predicted positive samples and the total number of positive samples. Below is the formula used to calculate the recall, where *F_N_* denotes the number of incorrectly predicted positive samples.

(11) $$R=\frac{T_{P}}{T_{P}+F_{N}}\times 100\%$$

Average Precision (*AP*): The value of average precision is equal to the area under the precision–recall curve. The calculation formula is presented as Equation (12).

(12) $$AP=\int_{0}^{1}P(R)dR$$

Mean Average Precision (*mAP*): The sum of average precision (*AP*) can be divided by the total number of classes (num_classes) to calculate the mean of the *AP* for each class (*mAP*). This metric of comprehensive performance is used to evaluate the model, reflecting its accuracy in identifying all categories. The method of *mAP* calculation is expressed as Equation (13).

(13) $$mAP=\frac{1}{N}\sum_{i=1}^{N}AP_{i}$$

where *N* represents the total number of classes, and denotes the average precision for the *i* class.

Aside from the aforementioned metrics of detection performance, this study also focuses on the computational complexity of the model. To assess this issue, the GFLOPs metric was introduced to thoroughly evaluate the model for its adaptability to the hardware platform.

### 4.3. Experimental Results and Analysis

#### 4.3.1. Analysis of Model Training Results

**Figure 8:**
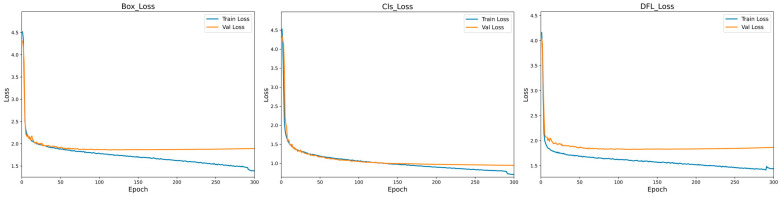
Loss function results plot.

Obviously, as indicated in Figure 8, 300 epochs were set during the training of the MS-YOLO model to ensure that the model could fully learn the features of the data and perform well in convergence. The loss functions of this model include Box_Loss, Cls_Loss, and DFL_Loss, all of which play a critical role in improving model performance. Among them, Box_Loss is used to measure the accuracy of bounding box predictions and indicate the degree of overlap between the predicted and ground truth boxes. Cls_Loss is used to evaluate how the model performs in classification. This is particularly significant to small object detection, and a reduction in Cls_Loss indicates overall model convergence. DFL_Loss is used to deal with the shape of complex or irregular objects, with its reduction indicating an improved model performance in processing the targets of various shapes. During the training phase, the loss functions are applied to guide the model toward the true values through weight adjustment. During the validation phase, the loss functions are used to evaluate the model performance on unseen data, for assessment of its generalization ability. Apart from indicating an improved model accuracy, the reduction in loss values also suggests that the model gradually converges during training, stabilizes, and ultimately achieves the optimal performance.

The Figure 8 shows the convergence of Box_Loss, Cls_Loss, and DFL_Loss during the training of the MS-YOLO model. Obviously, all the three loss functions exhibited a downward trend toward stable convergence during training, particularly in the first 50 epochs, where both training and validation losses converged rapidly. It is indicated that the model is effective in learning the target features in the early stage. As training progressed, the loss values stabilized, and the validation loss approached the training loss, indicating the excellent generalization performance of the model.

**Figure 9:**
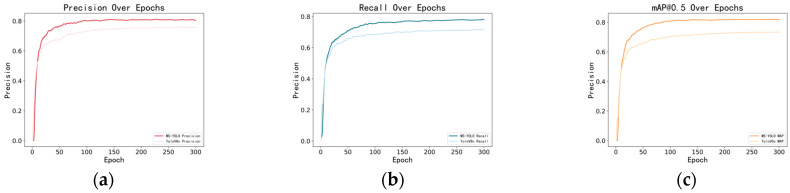
(**a**–**c**) show the precision and recall curves for YOLOv8n and MS-YOLO, as well as the comparison curve for mAP@0.5.

Comparison curves for precision, recall, and mAP@0.5 were plotted for YOLOv8n and MS-YOLO, with the gradual stabilization and improvement of each metric visualized during training. Relative to YOLOv8n, MS-YOLO performs much better in precision and recall. Additionally, it has a higher mAP@0.5 value, indicating an evident advantage of MS-YOLO in overall performance. See Figure 9.

#### 4.3.2. Ablation Experiments

As shown in Table 3, the various improvements in the proposed MS-YOLO model were evaluated. Specifically, these included (1) the BiFPN structure, (2) the MSI-SPPF feature fusion module, and (3) the MD-C2F and MDE-C2F modules, with the EMA mechanism integrated. Based on the experimental data used in this section, clear numerical indicators are provided for evaluating the performance of each improvement in the model.

In the MS-YOLO architecture, incorporating the BiFPN structure improved the performance of the model compared to the baseline, with both precision and recall being improved. The mAP@0.5 increased to 74.2%, demonstrating the effectiveness of the BiFPN module in enhancing the feature fusion performance of the network. When the MSI-SPPF module alone was introduced, all model metrics, particularly mAP@0.5 and F1, were significantly improved, indicating the important role played by this module in efficient feature extraction and fusion. When the MD-C2F and MDE-C2F modules were introduced, the GFLOPs were reduced to 7.0, but model performance was still improved, with the mAP@0.5 reaching 79.8%. It was confirmed that the lightweight design of these modules effectively reduces computational complexity while maintaining the high accuracy of detection.

Furthermore, when the BiFPN was combined with MSI-SPPF modules, the model performed significantly better, with a 5% increase in overall mAP@0.5. This illustrates the positive role of these two modules combined in feature extraction and fusion. When the BiFPN, MSI-SPPF, MD-C2F, and MDE-C2F modules were introduced simultaneously, the model achieved the optimal performance, with mAP@0.5 reaching 86.4%, a precision of 80.9%, and a recall of 77.8%. This evidences the potential applicability of these modules in combination to improve the overall model performance. Moreover, the GFLOPs of the model were reduced to 7.3, and the inference time was only 3.86 milliseconds according to the inference test conducted on the relevant hardware platforms, which is significantly advantageous over YOLOv8. These results indicate that the model achieves a significant improvement in accuracy while satisfying the requirement of hardware platforms for real-time inference.

#### 4.3.3. Comparison of Detection Results on the Self-Made Dataset

To evaluate the performance of MS-YOLO, tests were conducted on a self-built drowning dataset. Ablation experiments were performed to assess the importance of the MSI-SPPF, MD-C2F, MDE-C2F, and BiFPN modules, thus improving the understanding of their effect on model performance. Additionally, a comparison was drawn between the MS-YOLO model and other object detection algorithms, such as the Faster-RCNN, SSD, and the YOLO series. As indicated by the experimental results, MS-YOLO outperforms these popular algorithms in terms of accuracy.

The experimental results are shown in Table 4, where the performance of various models is compared in terms of precision, recall, mAP, F1 score, and model size. The two-stage detection model Faster-RCNN outperforms the SSD model in the accuracy of detection. However, it has a large model size which requires substantial computational power, which compromises real-time performance. The SSD model, as another single-stage detector, is outperformed by the YOLO series algorithms in model size, accuracy, and the speed of detection, which reaffirms the superiority of the YOLO series.

Regarding the YOLO series, all of them produce an excellent performance, particularly in precision and recall. YOLOv6n [42] achieves an mAP of 85.6%, illustrating a balanced performance on all metrics. However, in comparison, YOLOv9n and YOLOv10n perform slightly better in the accuracy of detection and F1 score, though the model size of YOLOv9n reaches 313.4 GFLOPs. Consequently, it is relatively more complex and less suitable for lightweight and flexible deployment.

**Figure 10:**
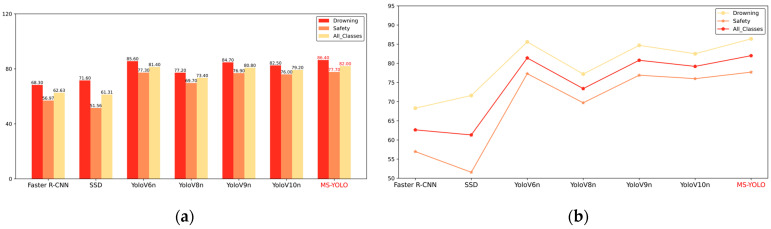
(**a**) is a bar chart, and (**b**) is a line chart. The results show that MS-YOLO achieves an accuracy of 86.4% in drowning detection, outperforming Faster R-CNN, SSD, and various versions of YOLO. The average detection accuracy is 82.0%.

**Figure 11:**
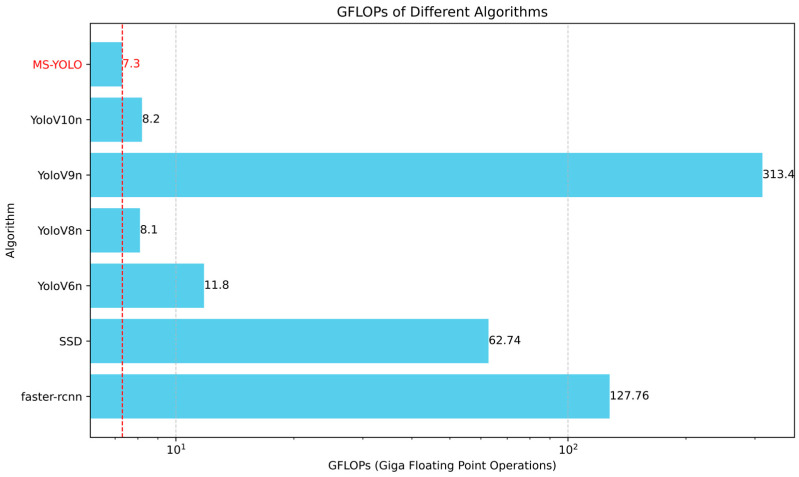
The GFLOPs of different detection algorithms are compared. MS-YOLO shows an exceptionally low level of computational load, with only 7.3 GFLOPs. It significantly outperforms other algorithms in this aspect. MS-YOLO reduces computational complexity substantially while maintaining high accuracy.

The MS-YOLO algorithm proposed in this paper significantly outperforms the existing algorithms in both the accuracy of detection and recall, with an mAP of 86.4%. It indicates a 2.6% improvement over YOLOv10 [43] and 1.6% over YOLOv9 [44], demonstrating the superior performance of the MS-YOLO model. Additionally, MS-YOLO achieves an F1 score of 82%, which further confirms its significantly improved performance in detection. Notably, the GFLOPs of the MS-YOLO model are reduced to 7.3, a 9.87% reduction compared to YOLOv8 [45]. Thus, the model becomes more lightweight while accuracy is effectively improved, which further validates the effective optimization by the MS-YOLO algorithm. See Figure 10 and Figure 11.

**Figure 12:**
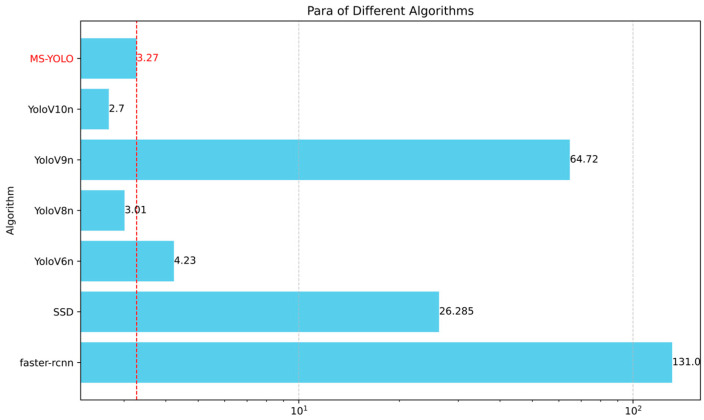
The number of parameters is compared between different detection algorithms. MS-YOLO stands out with a significantly lower parameter count of only 3.27 million, outperforming other algorithms in this regard. It reduces model size while maintaining high detection performance, which reflects its efficiency in balancing computational complexity with accuracy.

**Figure 13:**
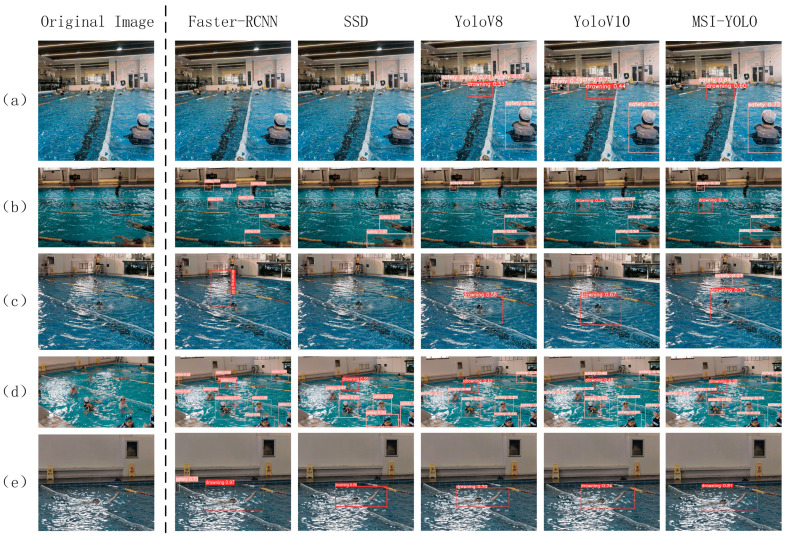
Defect detection results image. (**a**) Water-level view detection result, (**b**) aerial view detection result, (**c**–**e**) shoreline view detection results.

The prediction results were tested using five representative images. As shown in Figure 12, Faster-RCNN achieves high detection precision but is susceptible to missed detections and false positives. For instance, a single detection box in Figure 13d contains two different objects, indicating that it performs poorly in small object detection. Similar issues are encountered by the SSD model. Despite detecting most targets, YOLOv8 performs poorly in complex backgrounds, as reflected in Figure 13b. YOLOv10 performs well in various environments. However, it still performs worse than expected when compared to MS-YOLO in complex backgrounds. It also performs poorly when significant interference occurs. MS-YOLO performs much better in confidence level, with no missed or false detection. It produces an excellent outcome in small object detection, improving confidence and classification accuracy. See Figure 13.

## 5. Conclusions and Future Work

Herein, a novel and lightweight drowning detection model is proposed, namely MS-YOLO. By introducing the MD-C2F structure, the MDE-C2F module is developed to deal with the challenges in small object detection. Additionally, the advantages and disadvantages of SPP and SPPF are summarized, with MSI-SPPF proposed. Also, the bi-directional feature pyramid network (BiFPN) is incorporated by the model for rapid fusion and intra-channel sharing.

The results show that MS-YOLO achieves an accuracy of 86.4% in drowning detection and 77.7% in detecting safe swimming behavior. In comparison to YOLOv6, YOLOv9, and YOLOv10, the accuracy in drowning detection is improved by 0.6%, 1.2%, and 2.8%, respectively. Relative to YOLOv8, SSD, and Faster R-CNN, the accuracy increases by 8.6%, 15.4%, and 14.1%, respectively. Although YOLOv8 and YOLOv10 significantly outperform other models in computational load, MS-YOLO still reduces the load by 9.8%. MS-YOLO performs well across all metrics, which evidences its accuracy and reliability in drowning detection. Ablation experiments substantiated the advantages and effectiveness of the MS-YOLO model. According to the test results, the performance in drowning recognition is considerably improved by introducing the MD-C2F, MDE-C2F, MSI-SPPF, and BiFPN modules.

There is still room for future improvement in both the model and the custom-built dataset. The dataset used in this paper was collected indoors under normal illumination and water conditions, making it easier to identify the body movements and facial expressions of drowning individuals. However, it is necessary to take into account various unstable factors in outdoor environments, such as rainy or snowy weather, tides, and reflective ripples. Future work should focus on increasing the diversity of the dataset and deploying volunteers to collect data outdoors (e.g., at the beach, at outdoor swimming areas) with safety measures in place. Thus, the model can learn drowning characteristics in different complex environments. Additionally, drones, unmanned boats, and other devices can be used outdoors to capture images at different angles and heights for the model to better deal with the complex perspectives and variations in object size.

Moreover, in order to prevent the negative impact of complex conditions such as rain, snow, tides, and reflective ripples on drowning detection, noise reduction and light compensation, or dynamic environment improvements, are required for the detection model. By integrating visual signals with the data from other sensors (such as sonar and lidar), the limitations of computer vision in complex environments can be addressed. The detection model will be further improved in terms of weight for higher drowning detection accuracy, which is conducive to addressing the challenges in drowning rescue operations with less computational power required.

## Figures and Tables

**Table 1:** Experimental environment parameters.

Configuration Item	Version	Parameters
Operating System	Linux	
CPU	AMD EPYC 7542	42 cores
GPU	NVIDIA GeForce RTX3090	24 GB
Deep Learning Framework	Pytorch	2.2.1
Programming Language	Python	3.9
Computing Architecture	CUDA	12.1

**Table 2:** Training parameters.

Training Parameters	Values
Image size	640 × 640
Epochs	300
Batch	64
Works	16
Learning rate	0.01
Optimizer	SGD
Cache	True
Close music	10

**Table 3:** The impact of module placement within the backbone network.

BiFPN	MSI-SPPF	MD-C2F and MDE-C2F	P (%)	R (%)	MAP@0.5 (%)			F1 (%)	GFLOPs (G)
					Drowning	Safety	All		
			75.7	71.7	77.2	69.7	73.4	73	8.1
√			76	72.8	78	70.3	74.2	74	8.1
	√		78.5	73.9	82.4	73.8	78	76	8.7
		√	78.8	75.4	83.2	75.1	79.8	77	7.0
√	√		79.9	77.2	82.5	74.2	78.4	78	8.6
√	√	√	80.9	77.8	86.4	77.7	82	79	7.3

**Table 4:** Comparison results of different network models in drowning detection.

Algorithm	Precision (%)	Recall (%)	mAP@0.5 (%)			mAP@0.5:0.95 (%)	F1 (%)	GFLOPs (G)
			Drowning	Safety	All			
Faster-RCNN	64.8	70.2	68.3	56.97	62.63	28.9	67	170
SSD	84.61	61.24	71.06	51.56	61.31	28.4	71	31.4
YOLOv6n	78	76.1	85.6	77.3	81.4	36.1	78	11.8
YOLOv8n	75.7	71.7	77.2	69.7	73.4	31	73	8.1
YOLOv9n	78.2	72	84.7	76.9	80.8	36.1	78	313.4
YOLOv10n	77.3	75.6	82.5	76	79.2	34	76	8.2
MS-YOLO	80.9	77.8	86.4	77.7	82	36.5	79	7.3

## Data Availability

The data presented in this study are available upon request from the corresponding author. The dataset and code cannot be shared due to specific reasons.
